# The Influence of the Privatization Process on Accident Rates in the Forestry Sector in Poland

**DOI:** 10.3390/ijerph17093055

**Published:** 2020-04-28

**Authors:** Witold Grzywiński, Joanna Skonieczna, Tomasz Jelonek, Arkadiusz Tomczak

**Affiliations:** 1 Faculty of Forestry, Poznań University of Life Sciences, Wojska Polskiego 28, 60-637 Poznań, Poland; 2 Regional Directorate of the State Forests in Poznań, Gajowa 10, 60-959 Poznań, Poland

**Keywords:** accident incidence, fatality rate, forestry, public sector, private sector, privatization, Poland

## Abstract

The aim of this paper is to analyze the changes in accident rates resulting from the privatization of forest operations. Data from the years 1990–2017 were obtained from the Statistical Forestry Yearbooks issued by Statistics Poland, and were analyzed for two periods: the time of intensive privatization (1991–2002) and the post-privatization period (2003–2017). The data from 1990 were treated as a benchmark. There were 14,626 accidents in total, of which 236 (1.61%) were fatal. The non-fatal accident rate in the whole forestry industry showed a decreasing trend in the study period (t = 2.27, *p* < 0.05). In the case of the fatal accident rate we can observe an upward trend; in the period of intensive privatization the average annual fatality rate was 0.11, and after privatization it was 0.18 (t = −2.68, *p* < 0.05). In both periods the fatality rate was twice as high in the private forestry sector as in the public sector. The number of working days lost declined in the public sector and increased in the private sector. An accident in the private sector resulted in 20 days’ longer absence than one in the public sector. The study confirms that despite economic transition, accident rates in Polish forestry remain a serious issue. The main problem to be addressed is the increase in the fatal accident rate, especially in the private sector.

## 1. Introduction

According to the International Labor Organization definition, an occupational accident is defined as unanticipated and unplanned events that cause a personal injury, disease, or death [1]. The performance of work inevitably entails the possibility of the occurrence of accidents. Accident risk depends on many factors, including the type and number of occupational threats present in the working environment, as well as personal factors. Apart from human costs, occupational accidents also have enormous socioeconomic impacts around the world. Globally, over 264 million non-fatal occupational accidents requiring at least four days of absence from work occur annually, with 350,000 fatalities [2]. On a European scale, Eurostat reports that in 2017 there were over 3.3 million non-fatal accidents at work and 3552 fatal accidents in the EU-28 [3].

Some sectors of the economy are subject to particularly high accident risk. Forestry, commonly considered one of the most dangerous human occupational activities, belongs to this group [4,5,6,7,8]. This is especially visible in countries where the majority of forest operations are performed without the use of advanced technologies [9,10,11,12].

Because of their scale and their serious economic and social consequences, accidents in forestry have been the subject of many studies. However, most of these are focused on the analysis of accidents occurring during timber harvesting, which is an operation subject to the highest accident risk (e.g., [7,12,13,14,15,16,17,18,19,20]). Relatively few studies analyze accident occurrence on a wider scale, for example in a whole country, or compare accidents in the forestry sector against other industries [7,21,22,23].

The economic and social transitions in the countries of Central Europe after 1989 also altered the conditions in which the forestry sector functioned in those countries. They led to a significant reduction in employment in forestry, and the creation of the private forest services sector [21]. The privatization process, which began in Poland in the early 1990s, brought a gradual but almost complete transfer of logging and other forest operations to private companies providing forest services.

There is a common assumption that accident rates in forestry have decreased significantly in the last 10–20 years, especially in the countries of Central and Eastern Europe [21]. A similar view is taken with regard to forestry in Poland, especially in the public sector. However, long-term analyses covering the periods before and after the privatization of forest operations, using data from two of the 17 State Forests regional directorates, do not fully confirm this opinion [11,24].

The aim of this paper is to investigate long-term patterns in accident occurrence in Polish forestry and to analyze the changes in accident rates resulting from structural transitions, reduction of employment, and the inception of private forestry companies. Awareness of accident rates, the severity of accidents, and their circumstances and causes is essential for planning future actions to improve occupational safety in forestry.

## 2. Materials and Methods

### 2.1. Data

An analysis of accidents occurring in Polish forestry in the years 1990–2017 was carried out based on data obtained from the Statistical Yearbook of Forestry (SYF), published annually by Statistics Poland [25]. The limited range of information found therein meant that it was not possible to perform a full analysis of accidents. Some limitations also result from the system of data collection. A complete statistical breakdown between the private and the public forestry sectors has been made since 2000. Data related to numbers of employees are available from 1995, and data on days of incapacity for work from 1998. The data from 1990, before the privatization process had started, were treated as a benchmark.

On the basis of the available data, the following accident frequency and severity rates were calculated:

(1) Non-fatal accident rate—the number of accidents per 1000 employees;
(2) Fatal accident rate—the number of fatalities per 1000 employees;
(3) Non-fatal accident rate per production unit—the number of non-fatal accidents for each 1 million m^3^ of harvested timber;
(4) Fatal accident rate per production unit—the number of fatalities for each 1 million m^3^ of harvested timber;
(5) Number of lost working days—the total number of days of absence from work (excluding fatal accidents);
(6) Accident severity rate—the average number of days of incapacity for work confirmed by medical certificate.

### 2.2. Procedure

**Figure 1:**
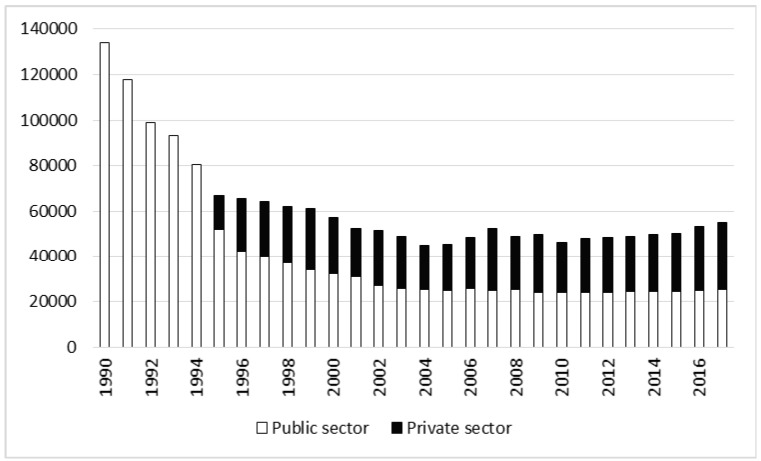
Number of employees in forestry in the years 1990–2017.

To determine changes in the occurrence of accidents in Polish forestry as a result of the privatization process, the 27 years of the study were divided into two periods: the time of intensive privatization (1991–2002) and the post-privatization period (2003–2017). In the second of these periods only a small number of manual workers remained in the public sector. The main criterion for delimiting the periods was the stabilization of employee numbers in the public forestry sector. The continuous decline in employment in the public sector (the State Forests), initially very rapid, began to slow toward the turn of the century and stabilized in 2003, which was considered as the end of the privatization process (Figure 1).

The statistical data concerning number of employees, volume of harvested timber, and non-fatal and fatal accidents were used to calculate the incidence rates of accidents. In turn, data concerning the number of accidents and days of incapacity for work were used to determine the severity rate.

### 2.3. Statistical Analysis

All statistical analyses were performed using the Statistica v. 11 software package (StatSoft Inc., Tulsa, OK, USA). To compare basic characteristics of accident incidence and accident rates, Student’s t-test for independent samples was used. Spearman’s rank correlation coefficient was used to establish the strength of trends in accident numbers and accident rates during the study period.

## 3. Results

### 3.1. Number of Accidents

**Figure 2:**
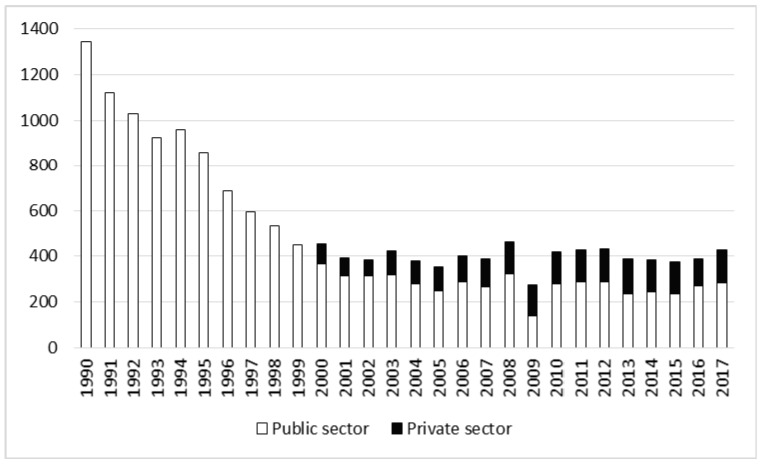
Number of non-fatal accidents in forestry in the years 1990–2017.

In the whole analyzed period of 27 years a total of 14,626 accidents occurred, of which 236 (1.61%) were fatal, and 480 (3.28%) were severe. The number of accidents was highest in 1991, and consistently decreased until the end of the 1990s. Since 1999 about 400 accidents per year have been recorded in the forestry industry (Figure 2). The number of non-fatal accidents in forestry as a whole gradually decreased over the study period (r = −0.70, *p* < 0.05) and differed between the privatization stages (t = 4.27, *p* < 0.05). Analysis by sector shows that accidents declined in the public sector and increased in the private sector; however, in the years 2003–2017 two-thirds of accidents still occurred in the public sector (Table 1 and Table 2).

**Figure 3:**
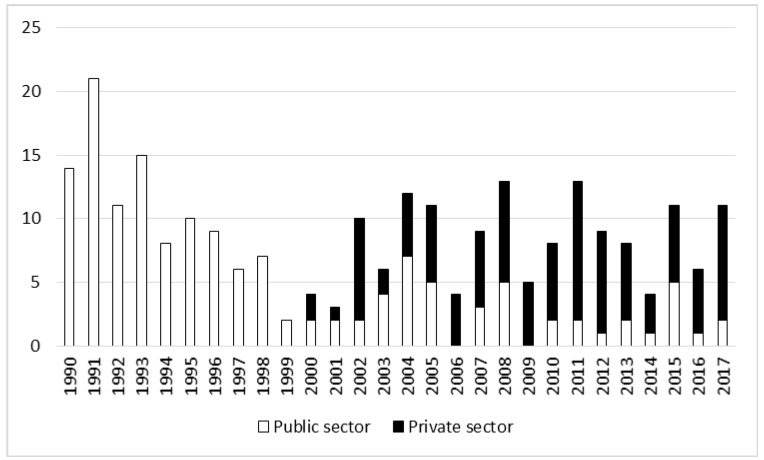
Number of fatal accidents in forestry in the years 1990–2017.

In 1990 there were 14 fatal accidents in the forestry industry, and in the privatization period the number of such events dropped to approximately 9 annually (Figure 3). There were no statistically significant changes in the number of fatal accidents between the studied periods (t = 0.10, *p* = 0.92) or in the whole analyzed period (Table 1 and Table 2). In both periods the fatality rate was twice as high in the private forestry sector as in the public sector (Table 1). In the years 2006 and 2009 no fatal accidents were recorded in the public sector (Figure 3).

### 3.2. Accident Incidence Rates

**Figure 4:**
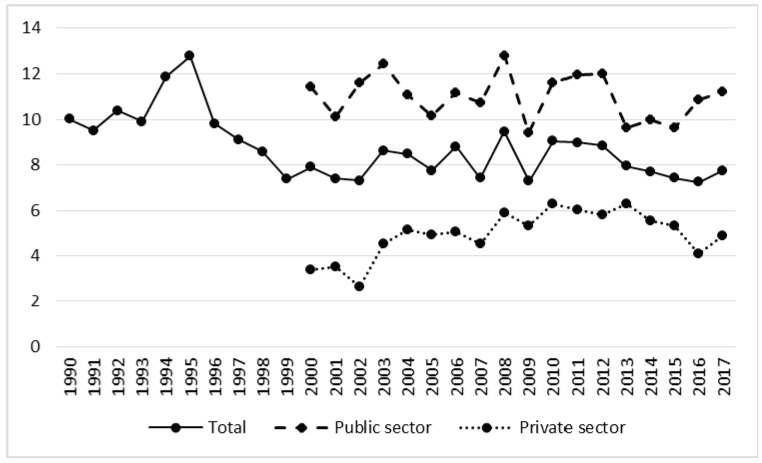
Rate of non-fatal accidents in forestry in the years 1990–2017.

The number of accidents does not reflect the actual level of accident risk, because its magnitude is strongly dependent on the number of workers. For the purpose of accident frequency analysis, non-fatal and fatal accident rates are used. In relation to forestry as a whole, the non-fatal accident rate was decreasing (t = 2.27, *p* < 0.05), despite its growth in the private sector in the post-privatization period (Table 1 and Table 2). The highest value was recorded in 1995 (12.92), and the lowest in 2016 (7.36) (Figure 4). Comparing the two periods we can observe a stable non-fatal accident rate in the public sector. Interestingly, accident incidence in the periods 1991–2002 and 2003–2017 was higher than in 1990.

**Figure 5:**
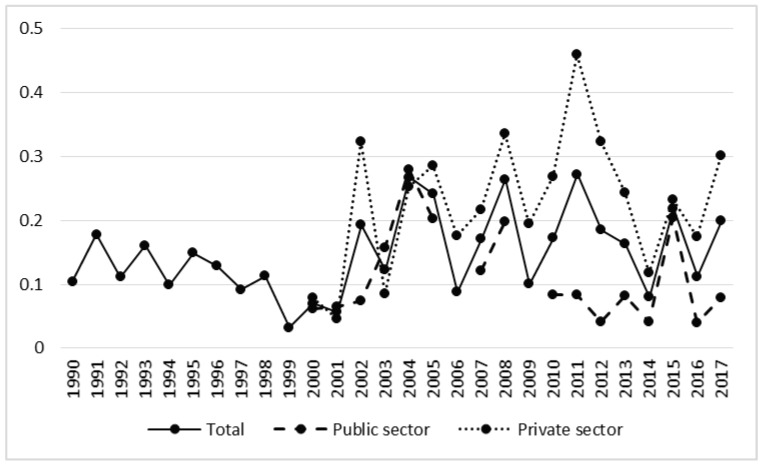
Rate of fatal accidents in forestry in the years 1990–2017.

In the case of the fatal accident rate we can observe an upward trend, especially in the private sector (Figure 5, Table 2). In 1990 the rate in both sectors was 0.10, whereas in the period of intensive privatization the average annual value was 0.11, and after privatization the average was 0.18 (t = −2.68, *p* < 0.05). The value of this indicator was twice as high in the private sector as in the public sector (Table 1).

**Figure 6:**
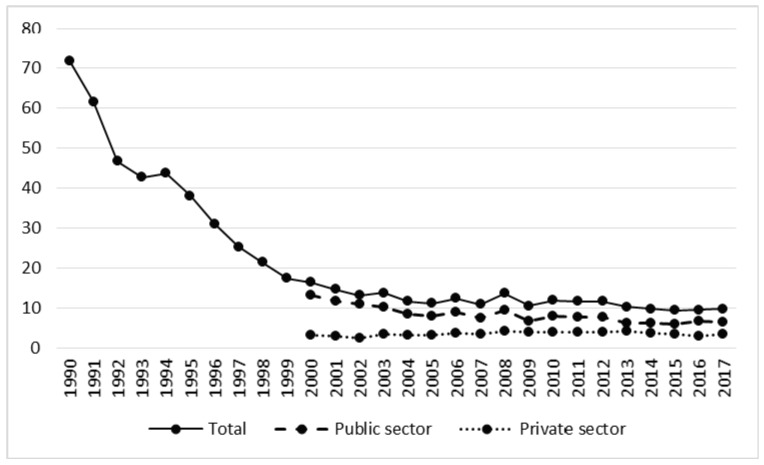
Rate of non-fatal accidents in relation to volume of harvested timber in the years 1990–2017.

The number of non-fatal accidents per 1 million m^3^ of harvested timber decreased significantly (r = 0.95, *p* < 0.05) (Table 2, Figure 6). In 1990 this figure was 71.91, in the years 1991–2002 it fell to 30.98, and in 2003–2017 it decreased further to 11.14 (t = −3.41, *p* < 0.05) (Table 1). This decline was strongly connected with a growth in the volume of timber harvested. In the post-privatization period the average volume of harvested timber was close to double the previous figure (23.8 vs. 36.3 million m^3^; t = −9.27, *p* < 0.05). The decline in the rate was observed in forestry as a whole and in the public sector; in the private sector a slight upward trend in the rate was recorded (Table 2).

**Figure 7:**
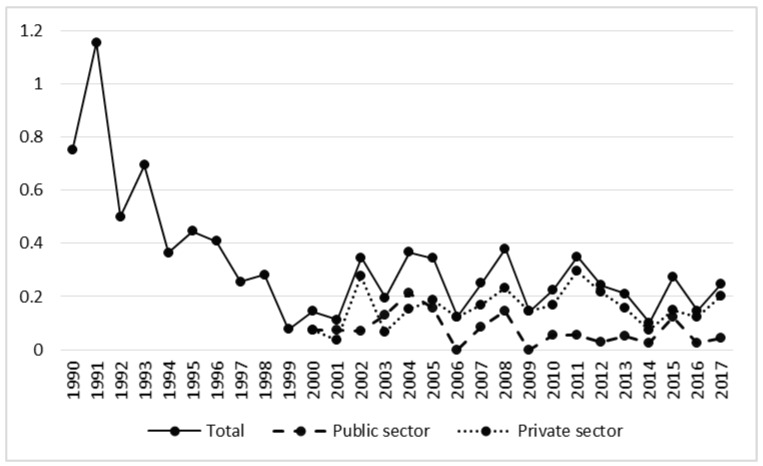
Rate of fatal accidents in relation to volume of harvested timber in the years 1990–2017.

The second production-based rate, the number of fatalities per 1 million m^3^ of harvested timber, also decreased, although not in the private sector (Table 2, Figure 7). This indicator averaged 0.40 in the privatization period and 0.24 in the post-privatization period (Table 1); however, this drop was not statistically significant (t = 1.97, *p* = 0.06). In the public sector the rate was the same in both analyzed periods (Table 1).

### 3.3. Accident Severity Rates

**Figure 8:**
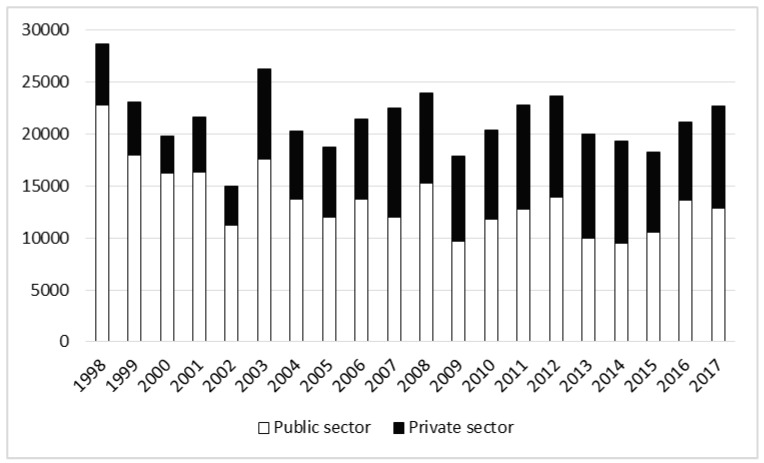
The number of working days lost in forestry in the years 1998–2017.

Accidents result in certain numbers of days of absence from work. We do not possess relevant data for 1990. Between 1991 and 2002 accidents resulted in 24,883 days of absence per year. In the subsequent period, time lost due to accidents fell to 21,303 working days annually (t = 1.45, *p* = 0.16), of which two-thirds were in the public forestry sector (Table 1). In the years 1991–2017 the total number of days of incapacity for work was 427,747, including 154,225 days in the private sector. The number of working days lost in forestry as a whole during the studied period decreased, but analysis by sector shows that accident severity declined in the public sector and increased in the private sector (Table 2, Figure 8).

**Figure 9:**
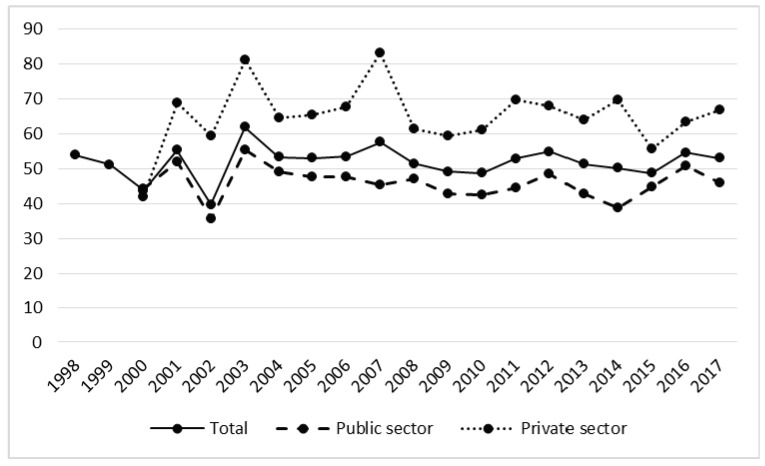
Severity rate of accidents in forestry in the years 1998–2017.

In spite of a decrease in working days lost in the years 2003–2017, the average length of sick leave in both sectors was subject to an undirected trend (Figure 9, Table 2). In the period of privatization an accident in the forestry industry caused an average of almost 49 days of sick leave, whereas in 2002–2017 this increased to 53 days, although this increase was not statistically significant (t = −2.03, *p* = 0.06) (Table 1). The average absence resulting from an accident in the private sector was 20 days longer than in the public sector.

### 3.4. Accident Causes

**Figure 10:**
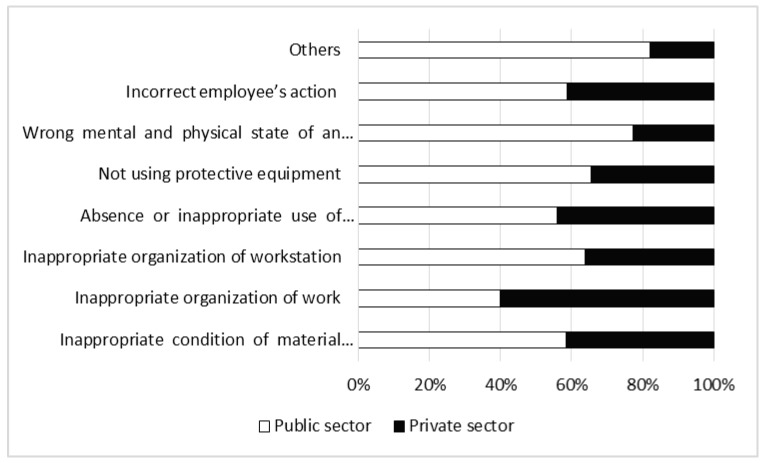
Causes of accidents in forestry in the years 1990–2017.

Over 60% of accidents in both sectors were caused by incorrect employee actions. Other causes were of much lesser significance. The next most frequent cause listed, inappropriate condition of material objects/agents, accounted for less than 10% of all accidents. The percentages attributed to other causes were similar in both sectors, except that the employee’s impaired psychophysical condition was more frequently identified as a cause in the public sector, and inappropriate organization of work was more frequent in the private sector (Figure 10).

## 4. Discussion

In Poland almost 7.6 million ha of a total 9.1 million ha of forests are owned by the state and are managed by the State Forests Holding. Privatization of the execution of forest operations in Poland contributed to a significant decline in employment in forestry, which also led to a decrease in the number of accidents in that industry. Despite this, forestry remains one of the most accident-prone sectors of the economy. The average accident rate in forestry in the years 2003–2017 amounted to 8.19 per 1000 employees, while for the country as a whole in 2017 the rate was 6.84 [26]. In turn, a study carried out in Slovakia for the period 2000–2016, where similarly to Poland the majority of forest operations are carried out by private companies, showed the absence of any significant drop in the accident rate in forestry [23].

The problem of high accident rates in forestry concerns many countries, including highly developed ones. Within the EU-28 in 2017, the agriculture, forestry, and fishing sector followed construction, administrative and support service activities, transportation and storage in the incidence rate of all non-fatal accidents at work. In 2017, agriculture, forestry, and fishing (12.8%) also had fourth place in terms of the total number of fatal accidents in the EU-28, and second place after mining and quarrying in the incidence of fatal accidents (6.1 per 100,000 persons employed) [3]. In the years 2000–2005, Gifford [21] estimated the number of fatalities in forestry in the EU at 24–30 annually. In New Zealand in the period 2006–2012 the average fatality rate in forestry was three times higher than in farming and ten times higher than in the economy as a whole [27]. In the United States in 2016 there were 91 fatalities in logging alone, and the fatality rate per 100,000 full-time equivalent workers amounted to 135.9 [28].

Surprisingly, there is a strong disproportion in the accident numbers between forestry sectors in Poland, with twice as many cases in the public sector. This is even more puzzling when we consider the fact that a significant number of employees in the public sector work in offices, where the accident risk is much lower than for field workers. Analyzing the accident rate in both sectors of forestry, we observe that it is decreasing slightly in the public sector, but consistently growing in the private sector.

The fatality rate was markedly higher in the private sector. The majority of fatal accidents take place during the timber harvesting process (e.g., [11,16,18,23,29,30,31,32,33,34]). The privatization of forest operations in the State Forests Holding in Poland resulted in the transfer of virtually all of the most dangerous operations to the private sector.

The non-fatal accident rate per 1 million m^3^ of timber production fell consistently in the whole period studied, from 71.91 in 1990 to 11.14 in the last years of the analysis. A rapid decline was recorded in the years 1991–1995, when the privatization process started. At the same time, the volume of harvested timber consistently increased. In 1990, 18.7 million m^3^ of timber was harvested; in the years 1991–2002 the annual average was 23.8 million m^3^; and in the last period of the study over 40 million m^3^ was produced every year.

The fatal accident rate per 1 million m^3^ also decreased, reaching a value of 0.24 in the last period of the study. A similar trend, resulting from increasing mechanization of logging and silvicultural operations, has been observed in other countries [27]. Ackerknecht [35] estimated forestry fatality rates in professional and legal operations for different countries in the years 2010–12. The fatality rate in Poland (0.24) is higher than the rates for Germany and Belgium (0.0 for each), Finland (<0.01), Sweden (0.04), New Zealand (0.16), Spain (0.18), the United States (0.22), and the United Kingdom (0.23), but lower than those for Austria (0.30) and Italy (2.05). There is probably also a link between the fall in the production-based fatal accident rate and an increase in the volume of timber harvested.

One of the consequences of accidents is absence from work on sick leave. In the last period of the study (2003–2017) the average annual number of days of incapacity was 30% higher in the public sector, partly because of a larger number of accidents reported in that sector. However, if we consider the severity rate, which indicates how many days were lost on average for a single accident, the situation is reversed. This rate indicates a higher number of severe accidents in the private forestry sector.

The severity rate increased in both sectors in the period after privatization to 46.3 days in the public sector and 66.9 days in the private sector. These values are very high compared with those reported in other studies. Tsioras et al. [18] estimated that the number of lost workdays because of accidents during timber harvesting in Austria ranged between 18.2 and 30.7, depending on the type and place of injuries. Even lower numbers of lost days were reported by Bentley et al. [15] in the case of New Zealand.

Regardless of the industry, accidents mostly occur because of employee fault [36,37]. So-called personal factors account for between one-third and two-thirds of incidents overall. The main cause of accidents is incorrect employee action, which means the failure to apply or intentional neglect of work safety rules and regulations. In Polish forestry this was the reason given for about 67% of all accidents, the figure being similar in both sectors. Other causes were much less frequent, and accounted for similar percentages of accidents in the two sectors.

Incorrect employee action may cause different effects in particular sectors of forestry. In the public sector, where the number of risk factors and level of danger are lower, the results of human error are mainly restricted to light injuries. In the private sector, especially during logging, any neglect of work safety may have serious health consequences. Therefore, accident prevention actions should be directed in the first instance toward private contractors.

## 5. Conclusions

This study confirms that accident rates in Polish forestry remain an important issue. The privatization of forestry operations in the State Forests Holding at the beginning of the 1990s merely transferred the problem from the public to the private forestry sector, rather than solving it. A slow and gradual decrease in the number of accidents is observed despite the increase in the volume of timber harvested. This is probably mainly due to the expansion of fully mechanized timber harvesting in the last 20 years [38,39]. From the example of such countries as Sweden and Finland, we know that mechanization is an effective and relatively rapid way to reduce the number of accidents in general, and fatal ones in particular [27].

The high and still growing fatal accident rate is alarming, especially in the private sector. The most dangerous activity in forestry is timber harvesting performed using the motor-manual method, which is still predominant in Poland. The Polish private forestry sector consists mainly of small enterprises whose only commercial activity is forestry. Additionally, low wages and piecework pay mean that contractors pay less attention to the observance of work safety regulations [20]. The aforementioned factors are responsible for the limited results brought by initiatives to promote health and safety in forest operations. For this reason, it is necessary to introduce a nationwide campaign on injury prevention, addressed to private contractors.

It seems that the best way to reduce the number of fatal accidents in the private sector of forestry is an increase in fully mechanized timber harvesting. If the motor-manual timber harvesting (chainsaws and skidders) is replaced with the fully mechanized method (harvesters and forwarders), the most hazardous factors in forest operations will be eliminated. Additionally, a well-organized system of recording of injuries, accident monitoring, extensive information about cases of accidents in forest operations, and trainings may also play a valuable proactive role. At the same time, the declining number of workers employed in forestry creates a good opportunity to take more determined action to improve the safety of forest workers.

## Figures and Tables

**Table 1:** Comparative analysis of number of employees and accident rates in forestry sectors in the years 1990–2017 (average values).

Forestry Sector	Number of Employees	Number of Non-Fatal Accidents	Number of Fatal Accidents	Non-Fatal Accident Rate	Fatal Accident Rate	Non-Fatal Accident Rate per Production Unit	Fatal Accident Rate per Production Unit	Number of Lost Days	Severity Rate
1990									
Public	134,000	1343	14	10.02	0.10	71.91	0.75	No data	No data
1991–2002									
Public	57,223 *	331.3 ^2^	2 ^2^	11.06	0.07	11.95 ^2^	0.07 ^2^	20,583.3 ^3^	43.96 ^3^
Private	23,267 ^1^	76.3 ^2^	3.67 ^2^	3.21	0.15	2.76 ^2^	0.13 ^2^	4300 ^3^	56.69 ^3^
Total	73,115 *	697.4 *	8.83	9.33 *	0.12 *	30.98 *	0.40	24,883.3 ^3^	48.75 ^3^
2003–2017									
Public	24,645 *	270.7	2.67	10.98	0.11	7.54	0.07	12,601	46.30
Private	24,652	130.7	6.00	5.32	0.24	3.60	0.16	8702	66.86
Total	49,135 *	401.4 *	8.67	8.19 *	0.18 *	11.14 *	0.23	21,303	53.02

* differences are statistically significant at *p* < 0.05; ^1^ data from 1995–2002; ^2^ data from 2000–2002; ^3^ data from 1998–2002.

**Table 2:** Spearman’s rank correlation coefficient of trends of accident characteristics in the years 1990–2017.

Characteristics	Public Sector	Private Sector	Total
Number of employees	−0.89 *	0.47 *	−0.68 *
Number of non-fatal accidents	−0.56 *	0.81 *	−0.70 *
Number of fatal accidents	−0.26	0.44	−0.10
Non-fatal accident rate	−0.23	0.53 *	−0.57 *
Fatal accident rate	−0.17	0.26	0.32
Non-fatal accident rate per production unit	−0.88 *	0.21	−0.95 *
Fatal accident rate per production unit	−0.60 *	0.34	−0.52 *
Number of lost days	−0.67 *	0.71 *	−0.30
Severity rate	−0.18	0.24	0.08

* differences are statistically significant at *p* < 0.05.
